# Nanocomposite hydrogels reinforced with vinyl functionalised silica nanoparticles

**DOI:** 10.1007/s10971-025-06989-x

**Published:** 2025-10-31

**Authors:** Ali A. Mohammed, Archontia Tsiampali, Siwei Li, Alessandra Pinna, Julian R. Jones

**Affiliations:** 1 Dyson School of Design Engineering, Imperial College London, London, UK; 2 Department of Materials, Imperial College London, London, UK; 3 School of Design, Royal College of Art, London, UK; 4 Visiting Specialist Services Academy Ltd, First Central 200, London, UK; 5 School of Veterinary Medicine, Faculty of Health and Medical Sciences, University of Surrey, Guildford, UK

**Keywords:** Hydrogels, Nanocomposites, Nanoparticles, Tissue engineering

## Abstract

This work reports double network hydrogel/silica nanocomposites with increased mechanical toughness and strength compared to their soft polymer-only counterparts. Applications are in tissue repair, such as cartilage, soft robotics and motion sensing. Covalent coupling of the sol-gel silica nanoparticles and the gel is vital because the gel swells on contact with water. Here, coupling was achieved through vinyl functionalisation of the silica nanoparticles (VSNPs) that enabled cross-linking to the network using photopolymerisation. The double network gel was an interpenetrating network hydrogel (IPNG) with 2-acrylamido-2-methylpropane-sulfonic acid (AMPS) as the first network, and acrylamide (AAm) as the second network. The effect of vinyl silica nanoparticle size and loading concentration were investigated on swelling behaviour, microstructure, compressive properties and nanoparticle retention. Increased size and loading concentration of VSNPs allowed for tailorability of swelling properties; nanocomposite IPNGs swelled less (88%) compared to control gels (97%). The nanocomposite IPNGs, with 20Wt% VSNPs, exhibited a max compressive strength of 810 ± 80 kPa at a strain of 75 ± 6%, similar to the lower range of articular cartilage, and an order of magnitude higher strength than control gels (90 ± 20 kPa, at a strain of 40 ± 3). SEM images show VSNP-polymer integration, with nanoparticles within the mesh walls. The nanocomposite structure provides reinforcement and toughness to soft IPNGs, making them suitable candidates for soft material repair.

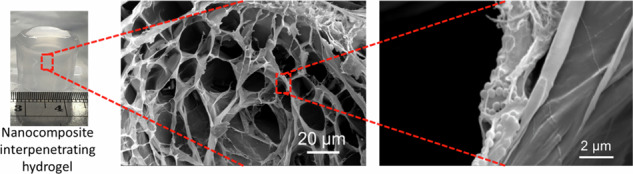

## Highlights

- Double network hydrogel/silica nanocomposites with increased mechanical toughness and strength compared to their soft polymer-only counterparts.
- Covalent coupling of the sol-gel silica nanoparticles and the gel reduce gel swelling and improve mechanical properties.
- Coupling was achieved through vinyl functionalisation of the silica nanoparticles (VSNPs) and photopolymerisation.

## Introduction

Hydrogels are water-swollen polymeric networks. They have potential in applications where swelling, due to water uptake, hydrophilicity and elasticity are beneficial, such as: biomedical applications, e.g. tissue regeneration, wound dressings, drug delivery systems; and soft robotics. The properties of hydrogels are dependent on parameters such as polymer structure and hydrophilicity, polymer molecular weight, cross-linking density, additives such as nanoparticles as fillers and intrinsic interactions between the 3D polymeric networks [1]. These parameters enable the tuning of both mechanical strength and water retention properties, but their mechanical properties are often low and insufficient [2–4]. Single network hydrogels have particularly poor mechanical integrity [5–8]. Attempts to improve the mechanical properties of gels have included multicomponent networks, also known as interpenetrating network hydrogels (IPNGs). IPNGs are a category of hydrogels that includes two or more polymers in the network in which the polymers are cross-linked with each other [9] and the networks cannot be separated until the chemical links are broken [10]. IPNGs can be synthesised in one-step or sequentially where the first network is synthesised first, and sequentially swollen in the second network to form the interpenetrating network. Double-network hydrogels (DNHGs) are a special class of IPNGs with much higher mechanical strength and fracture toughness [11]. DNHGs are tough and durable hydrogels with unique network structures composed of a tightly cross-linked rigid polyelectrolyte as the first network, and a sparsely cross-linked flexible neutral polymer as the second network [2, 4, 11–14]. The double networks result in superior mechanical properties, including a maximum fracture stress of ∼20 MPa [2, 15] at water content over 90%. The creation of DNHGs has shown that soft materials can achieve ductile, tough and strong properties whilst maintaining high water contents. Even with improvements using interpenetrating and double network systems, the limited mechanical stiffness of hydrogels creates drawbacks for the progression of applications such as cartilage repair due to mechanical fatigue and inability to recover from large deformations [16, 17].

Attempts have been made to increase the mechanical properties of IPNGs and DNHGs by making them into composites [2, 11, 18] through the incorporation of nanoparticles, such as Laponite clay, silica nanoparticles (SNPs), nanoceria, superparamagnetic iron oxide nanoparticles (SPIONs), carbon dots and cellulose nanocrystals [14, 19–24]. Although mechanical properties did increase, they were limited by poor fatigue and wear resistance.

A “super-tough” DNHG with covalently bonded vinyl-functionalised SNPs was developed by Fu et al. [14]. A conventional PAMPS/PAAm double network hydrogel was synthesised in the usual two-step polymerisation reaction using the thermal initiator potassium persulfate, initially forming the PAMPS network using MBAA. SNPs were added in the first network to form a PAMPS/SNP nanocomposite hydrogel. The SNPs behaved as macro cross-linkers in the hydrogel providing a denser and more covalently compact network. These nanocomposite hydrogels achieved a strain of 98% without fracture, a reported Young’s Modulus of 0.3 MPa. In that work, SNPs were 300 nm in diameter and a loading concentration of 1 wt% was used [14].

Another study showed that agar-PAAm DNHGs with graphene oxide (GO) nanoparticles can successfully be produced using a one-pot method [17]. The agar-PAAm/GO gels consisted of agar in the first network cross-linked via hydrogen bonds. PAAm was used as a second network with GO acting as physical cross-linking agent. These hydrogels exhibited mechanical properties with fracture strain of 4600% and fracture strength of 332 kPa. The GO infused nanocomposite hydrogels showed good fatigue resistance and self-healing ability.

Other similar work includes a thermoresponsive nanocomposite DNHG that was synthesised using N-isopropylacrylamide (NIPAAm) and 2 wt% of 50 nm and 200 nm polysiloxane nanoparticles. The resulting compressive stress reached a maximum of 175 kPa and 181 kPa, respectively, for 50 nm and 200 nm nanoparticles [25]. The incorporation of inorganic nanoparticles into the first network of DNHGs can be used to tailor the materials properties to achieve increased mechanical strength and toughness, improved resistance to strain and controllable swelling rates [4, 14]. SNPs are a suitable candidate as their surface contains -OH groups that can easily be chemically modified to change their functionality such as amine, vinyl or carboxylic groups [26, 27].

In this work, we aimed to use concepts of both IPNGs (sequential formation of networks) and DNHGs (use of contrasting polymers) to develop soft yet tough nanocomposite hydrogels using a photopolymerisation technique for both networks, as opposed to the conventional thermal polymerisation. The objective was to match the properties of articular cartilage as an exemplar gel-like material with good mechanical properties. Our strategy was to develop a nanocomposite structure using vinyl-functionalized silica nanoparticles (VSNPs) to provide photocurable groups that will covalently cross-link the first network consisting of 2-acrylamido-2-methylpropane sulfonic acid (PAMPS). We decided to swell the first network gel in a monomer solution of acrylamide (AAm) to produce the final nanocomposite hydrogels, with networks forming under free radical UV photopolymerization. The swelling and mechanical properties we were aiming for were; 70–80% water content at saturation and Young’s modulus 0.45–0.90 MPa [28]. Three different sizes of VSNPs (50, 100, 150 nm diameters) were tested with varying loading concentrations (0, 2.5, 10, 20 and 40 wt%) in the first network. AMPS and AAm were chosen due to their biocompatibility, hydrophilicity and relevance in soft material applications. The vinyl functional groups were chosen for the SNPs in order to form covalent cross-linkers with the monomers.

## Materials and methods

Ammonium hydroxide (NH_4_OH; 28–30% NH_3_ basis), tetraethyl orthosilicate (TEOS, 98%), ethanol (EtOH; 200 proof, anhydrous; ≥99.5%), acrylamide (AAm; ≥99%) 2-acrylamido-2-methyl-1-propanesulfonic acid (AMPS; 99%), N, N’-methylenebisacrylamide (BIS; 99%), photoinitiator 2-hydroxy-4′-(2-hydroxyethoxy)-2-methylpropiophenone (synonym Irgacure 2959; 98%), triethoxyvinylsilane (VTEOS; 97%) were purchased from Sigma-Aldrich (UK). No additional processing and/or purifications were performed.

### Silica nanoparticles synthesis

**Fig. 1:**
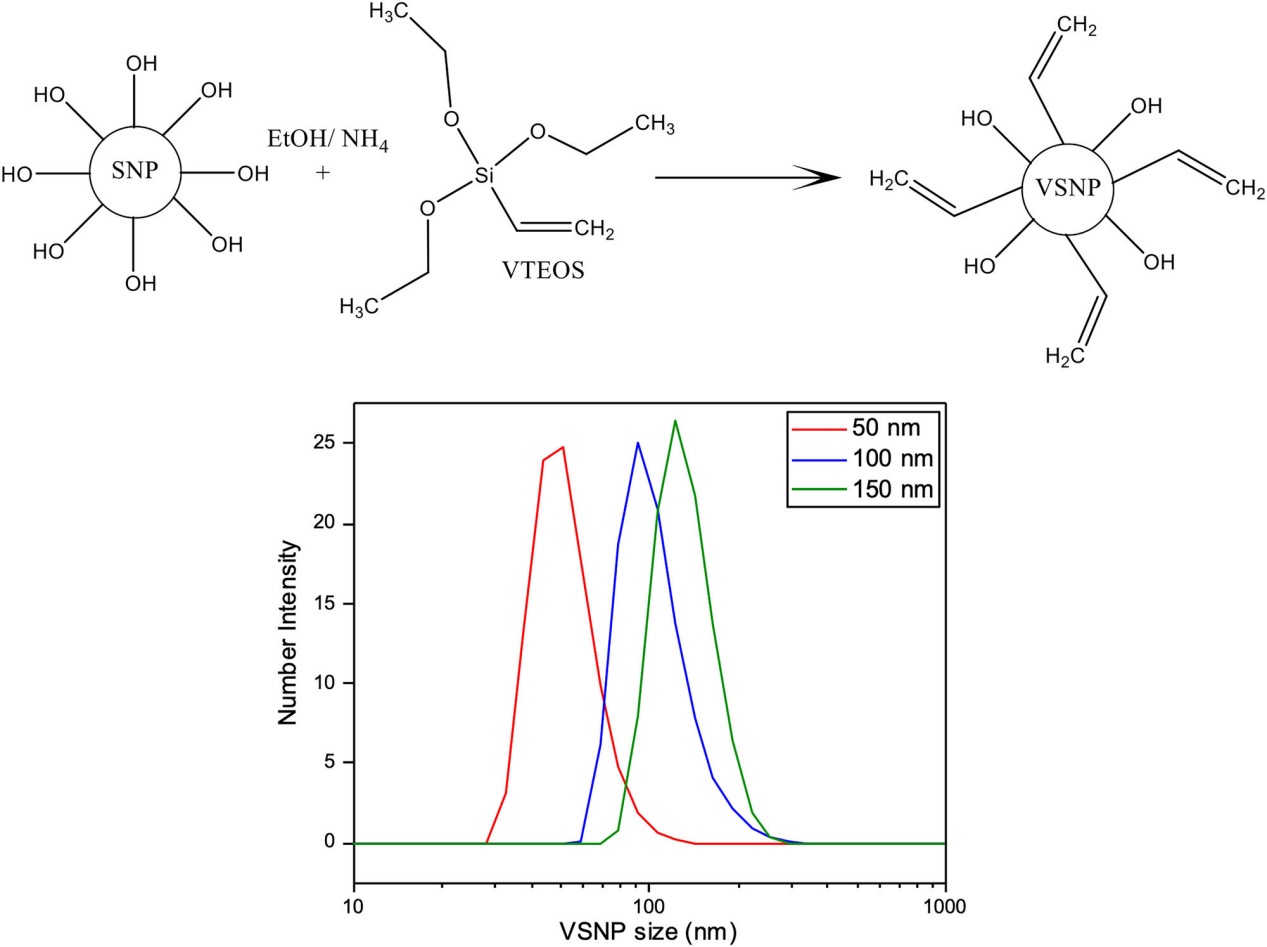
Surface functionalization of silica nanoparticles with VTEOS (top). Graph showing the size distribution of 50 nm, 100 nm and 150 nm vinyl silica nanoparticles (VSNPs) using DLS (bottom)

Silica nanoparticles (SNPs) were synthesized using a modified Stöber method to produce monodispersed SNPs [29–31]. SNPs with 100 nm diameter were synthesized by measuring out 82.33 ml EtOH and 10.30 ml (6 M) deionised water (DI-H_2_O) into a beaker. Whilst the mixture was stirring, 1.11 ml (0.28 M) NH_4_OH, were added to the solution followed quickly by 6.25 ml (0.28 M) TEOS as identified by Greasley et al. [30]. The solution was left to stir at 500 rpm for 24 h at room temperature. The final milky white solution was centrifuged using the Eppendorf 5430 Centrifuge and washed with EtOH three times at 7830 rpm to remove any unreacted TEOS. The final precipitate was left to dry at 60 °C overnight. The SNPs were then functionalised with vinyl groups by dispersing 500 mg of dried SNPs in 10 ml EtOH and sonicated using the CamSonix 1800T until a homogenous dispersion was achieved. The solution was then topped up with 90 ml EtOH whilst stirring. 500 µl anhydrous NH_4_OH and 3 ml vinyl triethoxyvinylsilane (VTEOS) were added to the solution and left to stir for a further 24 h. Once the reaction was complete, the solution was centrifuged and washed three times with EtOH to remove any unreacted VTEOS. The final precipitate was dried at 60 °C and stored under dry conditions. Figure 1 shows the surface functionalization chemistry of SNPs using VTEOS. 50 nm, 100 nm and 150 nm vinyl functionalized silica nanoparticles (VSNP) were formed and stored under dry conditions. VSNP sizes were confirmed using DLS (Fig. 1).

### Nanocomposite interpenetrating network hydrogel synthesis

0.48 M of AMPS and 1 wt% Irgacure 2959 were dissolved in 4 ml DI-H_2_O. 2.5 wt% of 50 nm VSNP (relative to AMPS mass) were sonicated in separate 1 ml aliquot in DI-H_2_O and added to the AMPS monomer solution whilst stirring. The AMPS/ VSNP mixture was pipetted into a 48-cell culture well and placed in the UV-Crosslinker for 5400 s with an average intensity of 2900 µW/cm^2^ of 365 nm UV light. Once the first network hydrogels were formed, they were taken out of the cell and prepared to be soaked in an AAm monomer solution.

1.41 M of AAm, 0.1 wt. % BIS and 1 wt. % Irgacure 2959 were dissolved in 5 ml DI-H_2_O. AMPS first network hydrogels were then swollen in the AAm monomer solution. Once fully swollen, the hydrogels were placed in the UV-Crosslinker for 5400 s under the same conditions as the first network. Once the second network was formed the hydrogels were dried at 60 °C and then placed in water for 240 h until a plateau was reached in their swelling state.

**Fig. 2:**
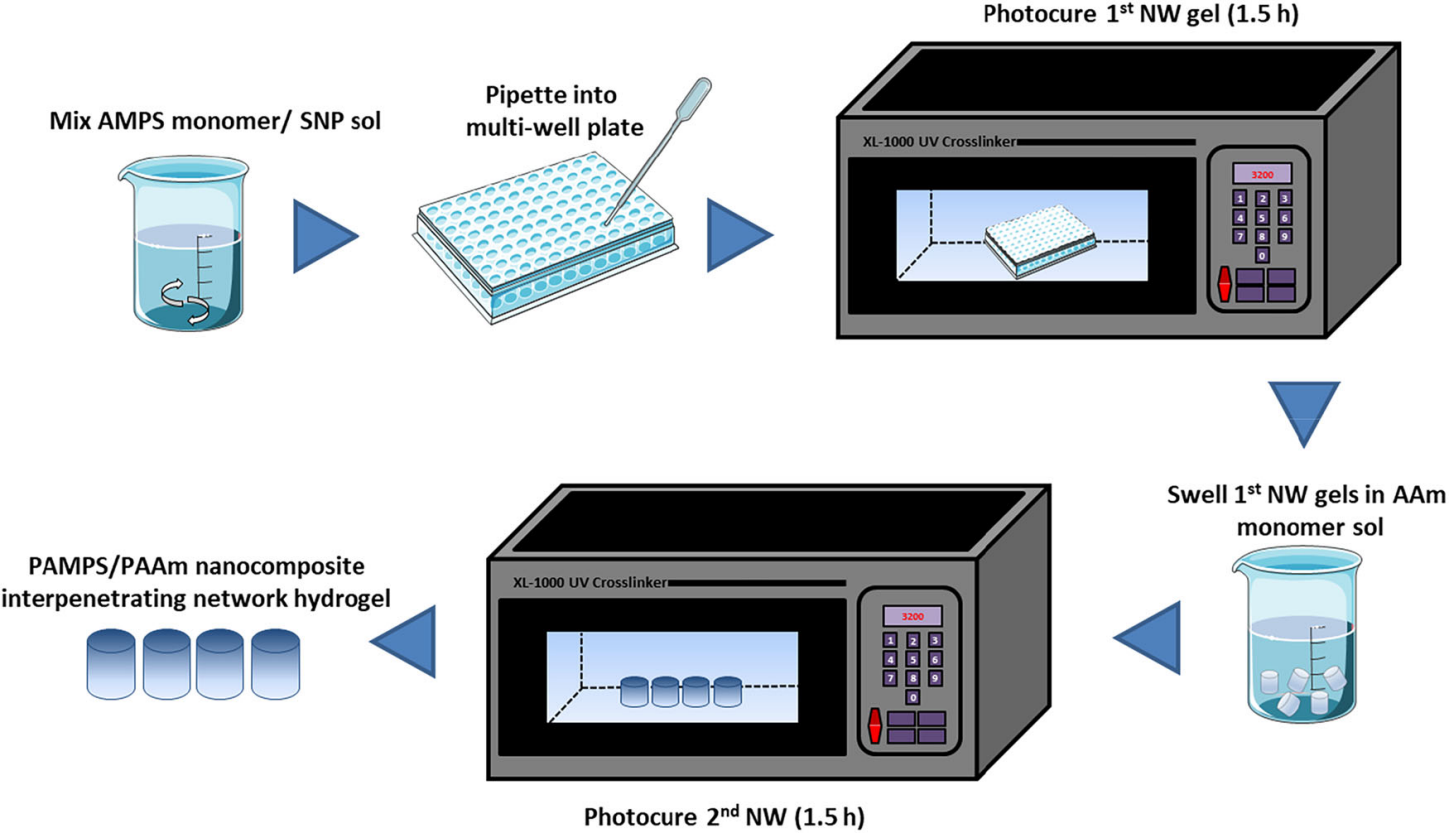
Synthesis route for PAMPS/PAAm interpenetrating network hydrogels containing vinyl silica nanoparticles (VSNPs)

This procedure was repeated IPNGs with 2.5, 10, 20 and 40 wt% VSNP loading for using 50, 100 and 150 nm VSNPs. A control IPNG without VSNP was formed under the same conditions, with 2.5 wt% BIS in the first network. The synthesis route is depicted in Fig. 2 below.

### Characterisation

Hydrogels were placed in DI-H_2_O post-synthesis and weighed at 0 h, 1 h, 2 h, 4 h, 8 h, 24 h, 48 h, 72 h, 120 h, 168 h and 240 h. Excess water was removed carefully using paper towels to ensure accurate mass readings. Swelling (%) was calculated using Eq. (1), and water content was calculated using Eq. (2):

1 $${\rm{Swelling}}\left( \% \right)=\frac{{{\rm{M}}}_{{\rm{s}}\left({\rm{t}}\right)}-{{\rm{M}}}_{{\rm{d}}}}{{{\rm{M}}}_{{\rm{d}}}}\times 100 \%$$

2 $${\rm{Water\; content}}\left( \% \right)=\frac{{{\rm{M}}}_{{\rm{s}}\left({\rm{t}}\right)}-{{\rm{M}}}_{{\rm{d}}}}{{{\rm{M}}}_{{\rm{s}}({\rm{t}})}}$$

Where M_s(t)_ is the swollen mass of the sample at time (t), and M_d_ is the initial dry mass of the sample.

TGA was carried out for all swollen samples to calculate nanoparticle retention (NPR) using Eq. (3). Swollen samples were dried for 5 days at 60 °C and ground to a powder. Polymeric material in the samples was expected to be burnt off by 600 °C and any remaining residues are considered to be SNPs.

3 $${\rm{NPR}}\left( \% \right)=\frac{{\rm{residual\; mass}}\left( \% \right)}{{\rm{theoretical\; mass}}\left( \% \right)}\times 100 \% =\frac{{\rm{residual\; mass}}( \% )}{{{\rm{M}}}_{{\rm{p}}}/{({\rm{M}}}_{{\rm{p}}}+{{\rm{M}}}_{1}+{{\rm{M}}}_{2})}\times 100 \%$$

Swollen samples were used for mechanical analysis in compression using a Zwick Roell z2.5 machine fitted with a load cell of 10 kN and a strain rate of 1.5 mm min^−1^.

DLS sample volumes of 100 µl were diluted in 1–1.5 ml of DI-H_2_O and sonicated using the CamSonix 1800T until the nanoparticles were suspended. Glass quartz cuvettes were used for analysis in the Nano ZS ZEN3600 Zetasizer, and each sample was run 3 times with 12 scans.

FTIR samples were dried at 60 °C and ground to a fine powder prior to analysis. A Nicolet iS10 Thermo Scientific FTIR was used. Samples were scanned 32 times per run with a resolution of 6 in a wavenumber region of 4000–400 cm^−1^.

Samples for SEM were frozen overnight at −80 °C and then freeze-dried for 24 h. Samples were placed on SEM sample holders and held by carbon tape. Samples were then sputter coated for 2 min at 20 mA to form a 10 nm layer of chromium. The LEO Gemini 1525 FEG-SEM was used to image the surface morphology of freeze-dried hydrogels using 3–5 kV and a working distance of 5–10 mm.

## Results and discussion

IPNGs with nanocomposite structures were successfully synthesized using VSNPs with varying sizes and loading concentrations. Samples without any post-processing are labelled ‘Fresh’ samples. IPNG hydrogels that were dried at 60 °C for 3 days and swollen for 10 days in DI-H_2_O referred to as ‘Swollen’ samples.

### Water content and swelling

**Fig. 3:**
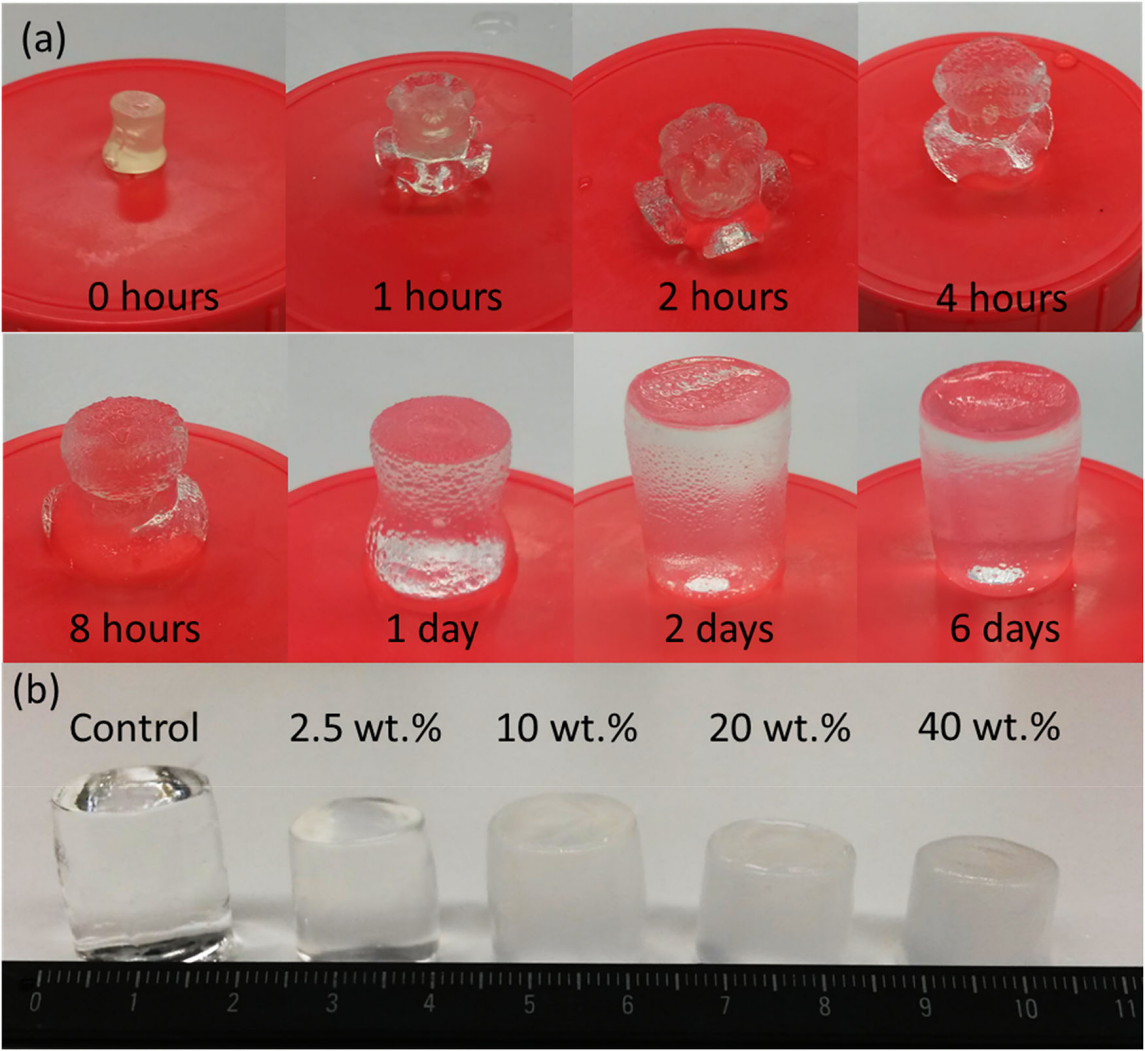
**a** Images showing interpenetrating network hydrogel (IPNG) containing 150 nm vinyl silica nanoparticle (VSNPs) at 2.5 wt% loading swelling behaviour from 0 h to 6 days in DI-H_2_O, and **b** from right to left: control IPNG, IPNGs containing 150 nm VSNP at 2.5 wt%, 5 wt%, 10 wt% and 20 wt% loading after 6 days of swelling in DI-H_2_O

**Fig. 4:**
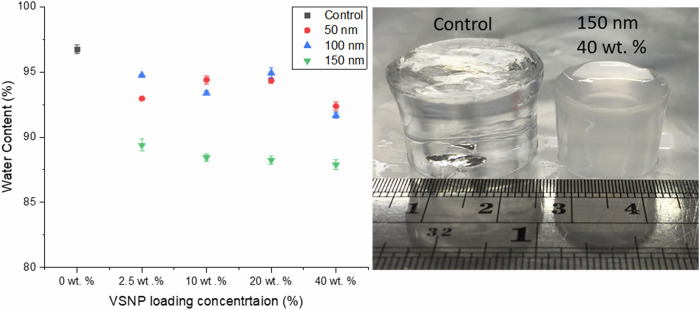
Graph showing water content of the interpenetrating network hydrogels (IPNGs) with 50 nm, 100 nm and 150 nm vinyl silica nanoparticles (VSNPs) at various loading concentrations after swelling for 6 days in DI-H_2_O, compared to a control with no VSNPs (left). Image showing a swollen control IPNG compared to an IPNG with 40 wt% of 150 nm VSNPs (right)

Samples were swollen in DI-H_2_O until a plateau in water uptake was achieved (≤2% change in volume). All IPNGs exhibited a ‘flowering’ pattern during the first 24 h of swelling, shown in Fig. 3. This is likely to be due to the difference in hydrophilicity of the two polymer networks, resulting in different rates of water uptake until both networks are fully saturated. Morphological factors such as cross-linking gradients or nanoparticle distribution may also contribute to this phenomenon. Figure 3b compares the nanocomposite IPNGs in ascending order of SNP loading. As VSNP loading concentration increased, the swollen size of the IPNGs decreased. Figure 4 provides a graph summarising the water content of the IPNGs and Table 1 provides a summary of water content and swelling values. The control gels exhibited a maximum water content of 96.7 ± 0.36% and swelling of 2757 ± 157%. Control gels plateaued at ~24 h, whilst the nanocomposite IPNGs plateaued closer to 72 h. Water content uptake decreased with the addition of VSNPs compared to the control. A reduction in swelling and swelling rate was observed when the first network was tightly cross-linked with VSNPs, compared to the control gel. No significant difference in swelling or water content was reported within samples of the same size at varying VSNP loading concentrations.

**Table 1:** Summary of the water content and swelling values of the interpenetrating network hydrogels (IPNGs) with 50 nm, 100 nm and 150 nm vinyl silica nanoparticles (VSNPs) at various loading concentrations, compared to a control with no VSNPs

	Loading concentration	Control	2.5 wt%	10 wt%	20 wt%	40 wt%
150 nm	Water content (%)	96.73% ± 0.36	89.39% ± 0.46	88.44% ± 0.30	88.24% ± 0.30	87.89% ± 0.38
	Swelling (%)	2757% ± 157	1287% ± 34	1168% ± 24	1320% ± 31	1220% ± 29
100 nm	Water content (%)	96.73% ± 0.36	94.76% ± 0.05	93.38% ± 0.19	94.93% ± 0.41	91.67% ± 0.24
	Swelling (%)	2757% ± 157	1811% ± 39	1415% ± 45	1890% ± 65	1695% ± 20
50 nm	Water content (%)	96.73% ± 0.36	92.96 ± 0.10	94.40 ± 0.32	94.35 ± 0.22	92.38 ± 0.33
	Swelling (%)	2757% ± 157	1321% ± 20	1686% ± 13	1673% ± 40	1218% ± 49

At 40 wt% loading of 150 nm VSNPs, water content was reduced by ~9%, to 87.9 ± 0.38%, compared to the control. 2.5 wt% VSNP loading in the first network had a similar effect on swelling as 40 wt% VSNP loading; this suggests the effectiveness of VSNP loading could be reached with as little as 2.5 wt%. The results are in line with previous work that demonstrated increased silica content above 1 wt% caused a reduction in the equilibrium swelling ratio due to the formation of a cross-linked rigid network [14]. VSNP cross-linking points in the hydrogel reduce the free volume in the hydrogels structure, reducing the swelling ability of the hydrogel [32].

IPNGs containing 50 and 100 nm VSNPs exhibited the greater water content across all samples, compared to IPNGs with 150 nm VSNPs. This is due to VSNPs with smaller diameters having less of an effect on the free volume in the network; therefore, water was still able to fill in the spaces resulting in more water uptake. Ultimately, an increase in both size and loading concentration will result in reduced water content and swelling. This is due to a combination of VSNPs acting as cross-linkers, void fillers or a combination. The extent of which they are either cross-linkers or void fillers will impact the mechanical properties and physical integrity of the hydrogel. Individual swelling profiles can be found in the Supplementary Information S1–3.

### FTIR

**Fig. 5:**
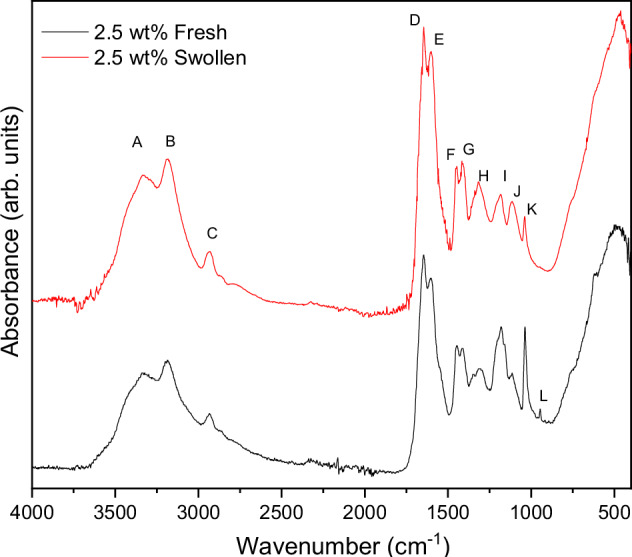
FTIR spectra comparing Fresh and Swollen PAMPS/PAAm hydrogels containing 2.5 wt. % 150 nm vinyl silica nanoparticles (VSNP)

Fresh and Swollen hydrogels containing 2.5 wt% of 150 nm VSNPs were dried and ground to a fine powder for FTIR analysis to test for any unreacted monomers (Fig. 5). Table 2 provides a summary of the bonds present and their respective bands present in the hydrogels, labelled (A-L). FTIR spectra showing all loading concentrations of 150 nm VSNPs for both Swollen and Fresh samples can be found in the Supplementary Information S4 and S5.

**Table 2:** Summary of FTIR bands for the PAMPS/PAAm interpenetrating network hydrogels

	Bond	Type	Wavenumber (cm^−1^)
A	N-H	Stretching	3324
B	O-H	Water remaining in sample	3082
C	C-H	Alkyl stretching	2950
D	C=O	Stretching, coupled with N-H bending	1652–1659
E	N-H	Primary amine	1620
F	C-N	Stretching primary amide	1420
G	C-H	Deformation	1350
H	S=O	Symmetric stretching	1317
H	N-H	Bending	1238
I	SO_2_	Symmetric stretching	1100–1250
I	Si-O-Si	Silica nanoparticles	1160
J	NH_2_	In-plane rocking	1120
K	R–S(=O)–R'		1040
L	C-H	Out-of-plane rocking	942

Bands found at 3324 cm^−1^ represent N-H stretching from PAMS and PAAm networks [13, 33]. 1652 cm^−1^–1659 cm^−1^ show C=O stretching coupled with N-H bending, that are attributed to both PAMPS and PAAm [13, 33]. 1620 cm^−1^ N-H primary amine deformation, 1350 cm^−1^ C-H deformation indicates the amine groups from acrylamide. 1420 cm^−1^ C-N primary amide stretching, 1350 cm^−1^ C-H deformation, and 1120 cm^−1^ NH_2_ in-plane rocking are present due to both polymers [13, 33]. Strong N-H stretching at 3324 cm^−1^ for PAAm was masked by OH, possibly due to incomplete dehydration of the sample, and as part of the sulfonic acid groups. Bands found at 2950 cm^−1^ represent alkyl vibrations of C-H bonds, from the presence of polymeric backbones [34, 35]. Sulfonic acid SO_2,_ –SO_3_H and S=O for PAMPS were found in the range 1100 cm^−1^–1250 cm^−1^, 1040 cm^−1^ and 1317 cm^−1^, respectively [33]. Unreacted monomer containing C-H bonds can be seen at 942 cm^−1^ and disappeared as VSNP loading increased, likely due to its lower abundance in the system. The Swollen spectra shows a noticeable reduction in intensity at 1040 cm^−1^ with the addition of VSNPs. This can be linked to broader band width associated with VSNPs covering the peak of sulfonic acid groups.

Figure 5 compares Fresh and Swollen samples of hydrogels containing 150 nm VSNP at 2.5 wt% loading. Unreacted monomers and unbound polymers were washed out of the samples during swelling, as seen in the reduction of intensity in the band regions 1000 cm^−1^–1500 cm^−1^. In particular, the reduction of symmetric stretching of SO_2_ at approximately 1100 cm^−1^–1250 cm^−1^, likely due to polymeric chains that are not cross-linked. A reduction in the 1120 cm^−1^ NH_2_ in-plane rocking allows the silica nanoparticle bands, Si-O-Si band at 1160 cm^−1^ [36], to become clearer. The remaining unreacted monomers are washed out from the Fresh hydrogel, as indicated by band L representing C-H out-of-plane rocking at 942 cm^−1^, when compared to the Swollen hydrogel.

### Nanoparticle retention

**Fig. 6:**
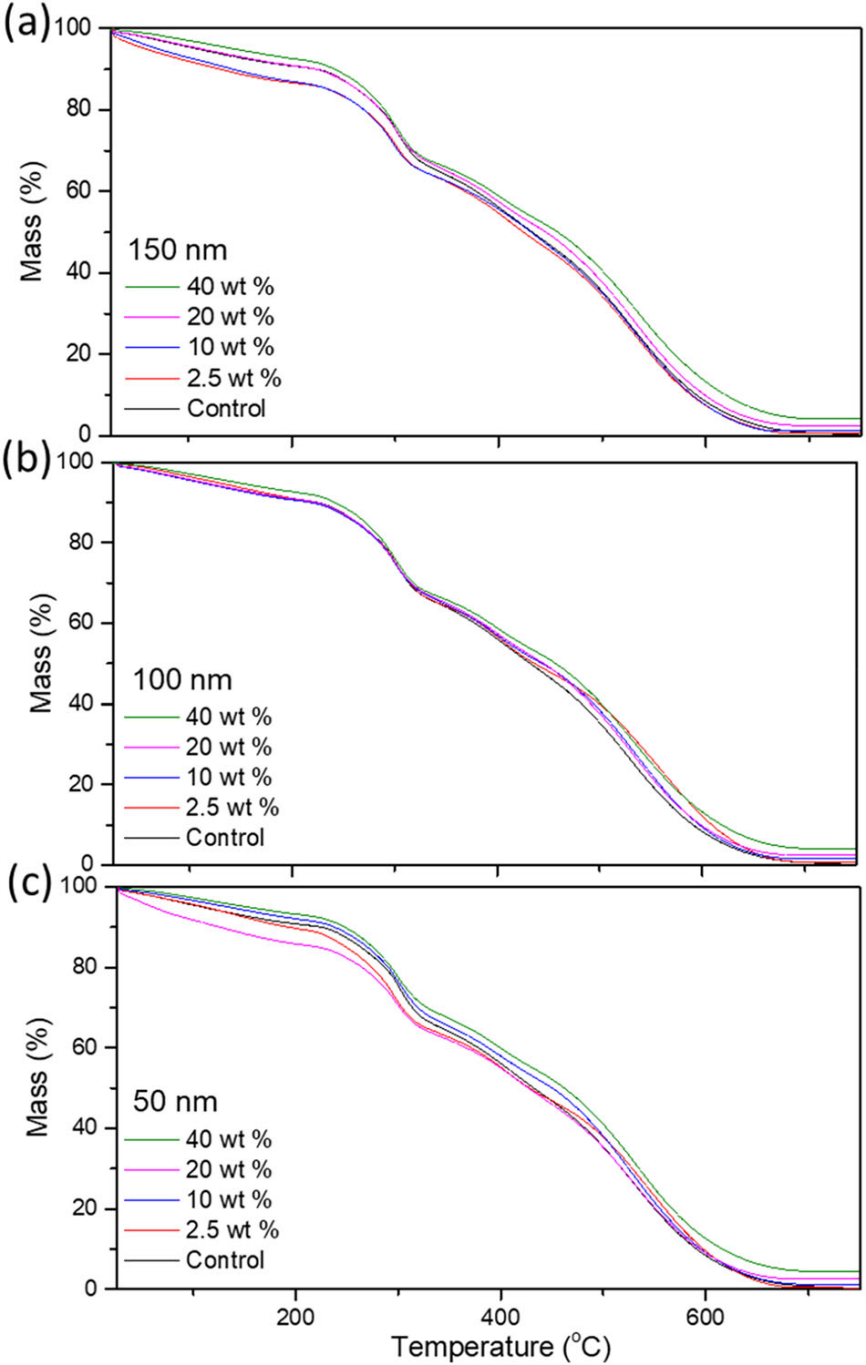
Thermal Gravimetric Analysis (TGA) of interpenetrating network hydrogels (IPNGs) with varying loading concentrations of 2.5 wt%, 10 wt%, 20 wt%, 40 wt% vinyl silica nanoparticles (VSNPs) compared to a control without VSNPs, with diameters of **a** 150 nm, **b** 100 nm and **c** 50 nm. TGA was run at a rate of 10 °C/ min up to 800 °C. Final mass values are summarised in Table 3

Figure 6 shows the TGA profiles of the IPNGs with varying VSNP loading concentrations with varying sizes of: 150 nm (Fig. 6a), 100 nm (Fig. 6b) and 50 nm (Fig. 6c). Individual TGA/ DSC graphs can be found in the Supplementary Information S6–8, which also show that control IPNGs result in 0% residual mass. Figure 6 shows the IPNGs followed a similar thermal degradation profile across all samples including the control. Previous TGA studies of AAm-co-AMPS copolymers revealed initial degradation of PAMPS at 182 °C and PAAm at 245 °C [37]. This suggests that PAMPS is less thermally stable than PAAm. There are three main degradation steps involved in the thermal decomposition of PAMPS/PAAm IPNGs: amide groups from PAAm, sulfonic groups from PAMPS, and the degradation of the polymeric backbones. The largest thermal degradation, reflected in the largest DTG peak, was noted in region between 250 and 300 °C and relates to the desulfonation of the material [38, 39]. TGA was used to calculate the NPR value using Eq. (3), assuming the final mass at 800 °C is the inorganic silica nanoparticles that have not burned off. Table 3 summarises the experimental NPR values compared to theoretical values. NPR values for gels with 2.5 wt.% VSNP loading were closest to the theoretical values whilst 40 wt% loading had the lowest NPR (Table 3). NPR was 100% for 2.5 wt% VSNP loading, but below 80% for the rest of the samples. Smaller VSNPs are more likely to percolate through structural pores while swelling the first network hydrogel in the monomer solution of the second network. This can be attributed to VSNP loss during swelling, uneven distribution of VSNPs due to aggregation and potential losses during synthesis due to VSNPs sinking to the bottom. DTG profiles (S6–8) reveal the first peak, representing desulfonation, was reduced in the presence of VSNPs. The DTG region between 350 and 450 °C shows more prominent peaks relative to the control, suggesting more energy was required to break bonds likely created by VSNPs in the first network. These graphs can be found in the supplementary information S6–8. Ultimately, results provided insight into the decreased retention at higher VSNP loading densities. NPR is important for the development of nanoparticle-loaded smart hydrogels. For example, stimuli-responsive magnetic soft robotics have incorporated iron oxide nanoparticles (IOPs) into filaments with tunable size within a gelatin methacryloyl hydrogels [40]. The size and concentration of the IOPs played a significant role in guiding cell alignment to the filaments. Another study integrated bioactive nanoparticles (BGNPs) into alginate dialdehyde-gelatin composite hydrogels [41]. BGNPs loading promoted the growth of a bone-like apatite layer on the surface of the hydrogels after they were immersed in a simulated body fluid. Other examples of hydrogels with specific functionality dependent on nanoparticles include magnetic hydrogel nanocomposites for remote controlled pulsatile drug release [42], auto-catalytic redox polymerisation using nanoceria for interpenetrating network hydrogels synthesis [3], and stimuli-responsive conductive nanocomposite hydrogels with self-healing for human motion sensing and wearable electronics [43]. Ultimately, this study shows a downtrend in NPR with increased VSNP loading concentrations.

**Table 3:** Summary of the experimental nanoparticle retention (NPR) values compared to theoretical values, for interpenetrating network hydrogels (IPNGs) with 50 nm, 100 nm and 150 nm vinyl silica nanoparticles (VSNPs) at various loading concentrations

	Loading concentration	2.5 wt%	10 wt%	20 wt%	40 wt%
	Theoretical %	0.42	1.67	3.33	6.67
150 nm	Residual %	0.40	1.57	2.43	4.08
	Particle retention %	96	94	73	61
100 nm	Residual %	0.42	1.30	2.32	4.25
	Particle retention %	100	78	70	64
50 nm	Residual %	0.33	1.24	2.55	4.41
	Particle retention %	79	74	77	66

### Mechanical testing

**Fig. 7:**
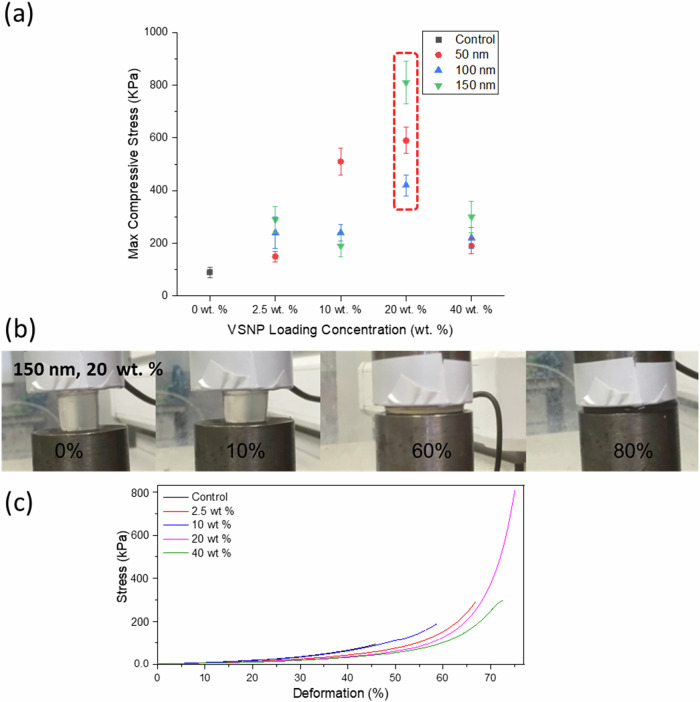
**a** Graph summarising maximum compressive stress values of interpenetrating network hydrogel (IPNGs) with 50 nm, 100 nm and 150 nm vinyl silica nanoparticles (VSNPs) at various loading concentrations, compared to a control with no VSNPs with a red dotted line highlighting the highest compressive values per VSNP size; **b** Images of a (IPNG with 20 wt% loading of 150 nm VSNPs under compression to ~80%; **c** graph showing the stress to strain profile of IPNGs with 150 nm VSNPs at 2.5 wt%, 10 wt% 20 wt% and 40 wt% compared to control

Swollen nanocomposite IPNGs were tested under compression until failure. Maximum compressive strengths are summarised in Fig. 7 and Table 4. Representative compression graphs for all samples can be found in the Supplementary Information S9–11. Figure 7a summarises the maximum compressive stress values of IPNGs with 50 nm, 100 nm and 150 nm VSNPs. Control hydrogels had a fracture compressive strength of 0.09 ± 0.02 MPa with 40 ± 3% deformation. Loading concentration of 2.5 wt% for 150 nm IPNGs produced a marked improvement in mechanical strength with a compressive strength of 0.29 ± 0.04 MPa and 67 ± 5% deformation. The addition of VSNPs provides strong physical adsorption between the PAMPS chains and VSNPs. This results in the improved mechanical strength due to energy dissipation through the desorption of PAMPS chains from the VSNPs [12, 19]. The lowest concentration therefore was effective at enhancing the compressive strength by three times and increasing the strain to failure by 27%. The largest improvement was noted at 20 wt% VSNP loading of 150 nm, with a compressive strength of 0.81 ± 0.08 MPa and 75 ± 6% deformation. Figure 7b shows a hydrogel containing 150 nm VSNP at 20 wt% loading under compression up to 80% before fracture. Figure 7c compares the stress to strain profile of hydrogels containing 150 nm VSNP at 2.5 wt%, 10 wt% 20 wt% and 40 wt% compared to control. Increasing the VSNP loading from 20 wt% to 40 wt% caused a decrease in mechanical properties across all VSNP sizes. This is likely due to overloading of VSNPs hindering the integrity of the IPNG. This could also be due to inhomogeneous distribution due to agglomeration or losses of VSNP during synthesis at 40 wt% as reflected by the respective NPR values. The increase in VSNP loading concentration led to decreased compressive stress and strain. This might be attributed to the increased rigidity of the first network having more cross-links and entanglements with the higher nanocomposite content, potentially limiting polymer chain flexibility leading to brittle behaviour. However, these values were still greater than the control IPNG. Reducing the size of the nanoparticles is hypothesised to produce larger specific surface areas for polymer-nanocomposite interactions, reflected in improvements using 50 nm VSNPs up to 20 wt%. Ultimately, compression studies revealed that improvements to the hydrogels provide impact with the lowest concentration of VSNP loading. Overloading of the first network was found to be in the region between 20 wt% and 40 wt% loading for all hydrogels, with the highest ultimate compressive strength found at 20 wt% VSNP loading for all three experimented sizes, as highlighted in Fig. 7a with a red dotted line. The combined concept and method used in this work can be used to tailor the mechanical properties of these hydrogels further by varying the concentration of the two polymer networks. These nanocomposite IPGNs are intended for cyclic applications such as cartilage repair or for bone tissue regeneration.

**Table 4:** Summary of compressive strengths of IPNGs with 50 nm, 100 nm and 150 nm vinyl silica nanoparticles (VSNPs) at various loading concentrations, compared to a control with no VSNPs

	Loading concentration	Control	2.5 wt %	10 wt %	20 wt %	40 wt %
150 nm	Fracture compressive stress (kPa)	90 ± 20	290 ± 50	190 ± 40	810 ± 80	300 ± 60
	Fracture strain %	40 ± 3	67 ± 6	60 ± 5	75 ± 6	73 ± 7
100 nm	Fracture compressive stress (kPa)	90 ± 20	240 ± 60	240 ± 30	420 ± 40	220 ± 40
	Fracture strain %	40 ± 3	61 ± 7	64 ± 4	72 ± 5	66 ± 7
50 nm	Fracture compressive stress (kPa)	90 ± 20	150 ± 20	510 ± 50	590 ± 50	190 ± 30
	Fracture strain %	40 ± 3	60 ± 4	73 ± 4	74 ± 6	65 ± 5

### SEM

**Fig. 8:**
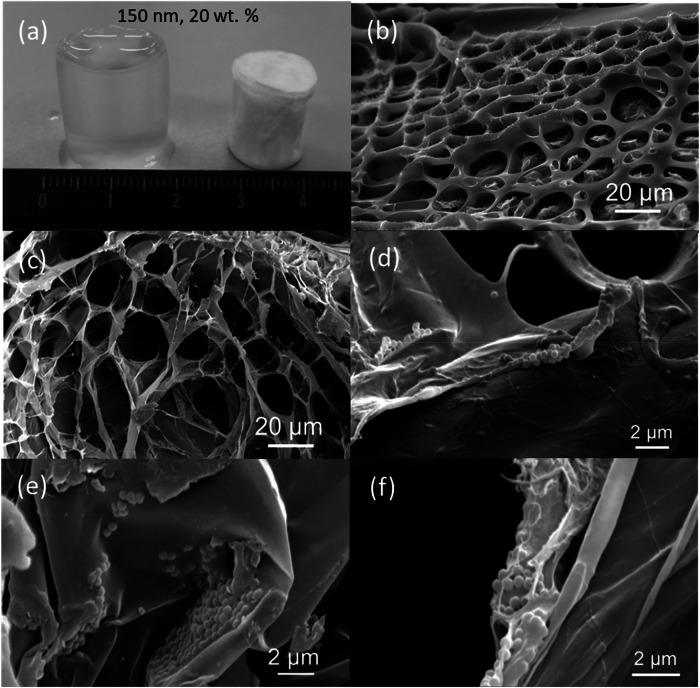
**a** Images of a swollen interpenetrating network hydrogel (IPNG) with 150 nm vinyl silica nanoparticles (VSNPs) at 20 wt% loading, compared to a freeze-dried IPNG of the same concentration; **b** SEM image of a control IPNG with no VSNPs; **c**–**f** SEM images of a IPNG with 150 nm VSNPs at 20 wt% loading at various magnifications showing the nanocomposite structure with the walls and channel struts

Hydrogel samples were frozen at −80 °C freezer overnight and then freeze-dried for 2 days. Figure 8a shows a IPNG with 20 wt% loading of 150 nm VSNPs, and a freeze-dried sample of the same composition. An average decrease in size of 24 ± 6% in diameter and 14 ± 5% in height was measured after the samples were freeze-dried. SEM images were taken for hydrogels containing 150 nm VSNP at 20 wt% loading and for the control, (Fig. 8b–f). Control hydrogels in Fig. 8b show web-like meshes with thick polymeric walls, typical of hydrogels after freeze drying [44, 45]. From observational analysis, mesh pore sizes were between 10 and 40 µm in the control samples and maintained a smooth circular pattern across the surface of the hydrogel.

Figure 8c–f shows thick polymeric walls which would explain enhanced strength with integrated VSNPs, compared to the control (Fig. 8b). Mesh pore sizes in the composite gels ranged between 10 and 50 µm with more elongated structures than in the control. Struts between pores and pore walls were examined to understand the integration and how the nanoparticles cross-link with the first network. VSNPs were found within struts and wall, Fig. 8d–f. This indicates that the VSNPs within the polymer matrix potentially act as cross-linkers and are well integrated into the gel mesh. The pores were examined and revealed that their polymer walls are infused with nanoparticles. This leads to the conclusion that the first network hydrogels retain the nanoparticles as a covalently cross-linked part of their structure. During compression and tension, the nanoparticles displayed in Fig. 8d–f are likely to hold the material together and act as physical reinforcements to withstand damage and resist breakage. Thus, clarifying the method in which strain is improved in VSNP loaded hydrogels compared to control hydrogels.

## Conclusion

Nanocomposite interpenetrating network hydrogels were successfully synthesised by a combination of methods of interpenetrating networks and double network hydrogels, using vinyl-functionalised silica nanoparticles as cross-linkers with PAMPS in the first network. The effect of the size of the nanoparticles, and the loading concentration in the first network were examined. PAMPS polymer chains were cross-linked by the nanoparticles resulting in an internal micro-network that was able to dissipate the compressive stresses, resulting in improved stress and strain values. A critical concentration was reached at 20 wt% for the hydrogels synthesised in this work where a marked improvement was exhibited. The combination of size and loading density of 150 nm at 20 wt% was deemed the most effective, with a maximum compressive strength of 810 kPa and strain of 75%. However, overloading occurred at 40 wt% resulting in decreased mechanical integrity, suggesting the nanocomposites begin to hinder the internal structure. SEM images revealed the interaction between nanoparticles and polymeric mesh, providing insight into the potential mechanism in which the nanoparticles help sustain increased compressive strains. IPNGs exhibited lower nanoparticle retention with increased loading densities above 2.5 wt%, likely due to inhomogeneous mixing or a sinking effect during synthesis leading to losses during synthesis. The IPNGs also demonstrated substantial swelling capacity, with water content ranging from 88 to 97%, depending on VSNP size and concentration. Ultimately, a tailorable nanocomposite interpenetrating network hydrogel was formed using various sizes and concentrations of nanoparticles as cross-linkers. The compressive strength and water content of this material prove to be ideal for soft tissue engineering, as they demonstrate mechanical properties akin to those of soft tissues and articular cartilage. These hydrogels possess the capability of incorporating bioactive substances, such as growth factors or stem cells, which enables the creation of versatile biomaterials. These multifunctional hydrogels hold the potential to not only foster the bonding of the scaffold to the host cartilage or bone but also to encourage the development of new tissue formation.

## Supplementary information

Supplementary information

## Data Availability

The data that support the findings of this study are available from the corresponding author upon reasonable request from rdm-enquiries@imperial.ac.uk. For the purpose of open access, the author has applied a Creative Commons Attribution (CC BY) licence (where permitted by UKRI, ‘Open Government Licence’).
