# Supplementary material for: Nanocomposite hydrogels reinforced with vinyl functionalised silica nanoparticles
Source: J Solgel Sci Technol. 2025 Oct 31;116(2):861–73. doi: 10.1007/s10971-025-06989-x (PMC12662862; doi:10.1007/s10971-025-06989-x)
Supplement: Supplementary file 1 — Supplementary information [file 10971_2025_6989_MOESM1_ESM.docx]

**Nanocomposite hydrogels reinforced with vinyl functionalised silica nanoparticles**

Ali A. Mohammed ^1, 2, 3^, Archontia Tsiampali ^2^, Siwei Li ^2,4^, Alessandra Pinna ^2, 5, 6^, Julian R. Jones^2, *^

^1^ Dyson School of Design Engineering, Imperial College London, SW7 9EG

^2^ Department of Materials, Imperial College London, SW7 2AZ, London, UK

^3^ School of Design, Royal College of Art, SW11 4AY, London, UK

^4^ Visiting Specialist Services Academy Ltd, Office 6.072 6th Floor, First Central 200, 2 Lakeside Drive London NW10 7FQ

^5^ The Francis Crick Institute, London NW11 AT, UK

^6^ School of Veterinary Medicine, Faculty of Health and Medical Sciences, University of Surrey, Guildford GU2 7XH, UK

Supplementary Figure 1. Swelling profile for PAMPS/PAAm hydrogels with 150 nm vinyl silica nanoparticles (VSNPs) at different loading concentrations compared to control.

Supplementary Figure 2.- Swelling profile for PAMPS/PAAm hydrogels with 100 nm vinyl silica nanoparticles (VSNP) at different loading concentrations compared to control.

Supplementary Figure 3.- Swelling profile for PAMPS/PAAm hydrogels with 50 nm vinyl silica nanoparticles (VSNP) at different loading concentrations compared to control.

Supplementary Figure 4. FTIR spectra for Fresh PAMPS/PAAm hydrogels with 150 nm vinyl silica Hydrogels containing 150 nm VSNP were freeze dried and investigated under SEM.

Supplementary Figure 5. FTIR spectra for Swollen PAMPS/PAAm hydrogels with 150 nm vinyl silica nanoparticles (VSNP) at different loading concentrations compared to control.

Supplementary Figure 6. TGA and nanoparticle retention for PAMPS/PAAm hydrogels with 150 nm vinyl silica nanoparticles (VSNP) at different loading concentrations compared to control.

Supplementary Figure 7. TGA and nanoparticle retention for PAMPS/PAAm hydrogels with 100 nm vinyl silica nanoparticles (VSNP) at different loading concentrations compared to control.

Supplementary Figure 8. TGA and nanoparticle retention for PAMPS/PAAm hydrogels with 50 nm vinyl silica nanoparticles (VSNP) at different loading concentrations compared to control.

Supplementary Figure 9. Compression curves for PAMPS/PAAm hydrogels with 150 nm vinyl silica nanoparticles (VSNP) at different loading concentrations compared to control.

Supplementary Figure 10. Compression curves for PAMPS/PAAm hydrogels with 100 nm vinyl silica nanoparticles (VSNP) at different loading concentrations compared to control.

Supplementary Figure 11. Compression curves for PAMPS/PAAm hydrogels with 50 nm vinyl silica nanoparticles (VSNP) at different loading concentrations compared to control.
